# Telomeres, Telomerase, and Curcumin: A New Frontier in Cancer Therapy: A Narrative Review

**DOI:** 10.3390/biomedicines13112721

**Published:** 2025-11-06

**Authors:** Hind Muteb Albadrani, Abeer Fouad Zakariyah

**Affiliations:** 1 Department of Clinical Laboratory Sciences, College of Applied Medical Sciences, Imam Abdulrahman Bin Faisal University, Dammam, Saudi Arabia; hmalbadrani@iau.edu.sa; 2 Department of Basic Medical Science, Division of Medical Genetics, College of Medicine, University of Jeddah, Jeddah, Saudi Arabia

**Keywords:** cancer, curcumin, telomere, telomerase

## Abstract

Telomeres, which serve as protective ends on chromosomes, and telomerase, the enzyme that preserves telomere length, play crucial roles in ensuring genomic stability and delaying cellular aging. Dysregulation of these proteins is a key characteristic of cancer development. This review aimed to explore the complex processes involved in telomere and telomerase dysregulation in cancer and evaluate the therapeutic potential of curcumin. Curcumin has attracted significant interest due to its anticancer, antioxidant, and anti-inflammatory properties. Curcumin modulates telomere dynamics and inhibits telomerase activity, leading to cancer cell senescence and telomere shortening. Curcumin downregulates human telomerase reverse transcriptase expression and reduces telomerase activity in various cancer cell lines. Despite its potential, its clinical use is restricted by its poor water solubility and limited bioavailability. This review underscores the critical role of telomere/telomerase dysregulation in cancer and highlights curcumin as a promising modulator of these pathways, thereby offering potential novel strategies for cancer treatment. This review integrates the literature published up to September 2025 to ensure the inclusion of the most recent advances in curcumin-related telomerase modulation.

## 1. Introduction

Telomeres are repetitive DNA-protein that protect the ends of chromosomes and help preserve their genomic stability and integrity. Telomerase is a ribonucleoprotein enzyme complex that prevents telomere shortening by adding repeated DNA sequences to the ends of telomeres during cell division. Dysregulation of telomeres and telomerases has implications in cancer initiation and progression [1]. Telomeres become shorter with each round of cell division in normal cells until they reach a point where the cell either stops dividing or undergoes apoptosis, thereby serving as a safeguard against uncontrolled cell proliferation. Telomerases play an important role in maintaining telomere length, which is essential for cellular longevity and stability. Under normal physiological conditions, telomeres become shorter with each round of cell division, leading to cellular aging and eventual senescence [2]. However, telomerase counteracts this shortening by adding repetitive nucleotide sequences to telomeres, thereby extending their length and allowing cells to divide beyond the typical limit. This activity is particularly important in germ cells (sperm and eggs) and undifferentiated stem cells, such as the skin, intestine, and hematopoietic system, where maintaining telomere length is vital for tissue regeneration and reproductive capacity.

In cancer cells, the mechanisms that maintain telomere length can overcome the replicative limit, allowing them to proliferate indefinitely and achieve an immortal status. Cancer cells with dysfunctional telomeres contribute to chromosomal instability and are involved in oncogenic transformation, metastasis, and resistance to therapy [3,4]. Therefore, the complex dysregulation of telomeres in cancer and telomerase activity highlights the necessity of developing therapeutic interventions based on an understanding of these mechanisms. The telomer elongation pathway regulates cancer growth, and targeting this pathway has significant therapeutic potential for preventing the growth and progression of most cancers. Curcumin, a polyphenol derived from the rhizome of *Curcuma longa*, has garnered significant research interest due to its diverse pharmacological characteristics, including anticancer, antioxidant, and anti-inflammatory effects [5].

Curcumin modulates telomere and telomerase dynamics, offering potential therapeutic benefits in cancer treatment. Curcumin blocks telomerase activity by downregulating various telomerase components, including human telomerase reverse transcriptase (hTERT), in various malignant cell lines [6]. Curcumin also shortens telomere length and triggers senescence-like growth arrest in several cancer cells, thus reducing their potential to proliferate. It also exhibits powerful antioxidant activity, which may reduce the telomere damage caused by oxidative stress, a characteristic feature of many cancer cells. The multidimensional effects of curcumin on the telomere/telomerase pathways hold promise for the development of new therapeutic strategies in cancer management [5].

Considering the crucial role of telomeres and telomerase dynamics in cancer development, along with new evidence that curcumin can influence these pathways, this review provides a foundation for future studies. This highlights the promise of curcumin as a new therapeutic agent and suggests new directions for creating targeted cancer treatment that takes advantage of its diverse benefits. Although the anticancer properties of curcumin are well documented, its modulatory effects on telomere and telomerase dynamics in cancer remain unclear. This literature review aimed to explore the complex interactions between telomeres, telomerases, and cancer development, focusing on understanding the mechanisms underlying the deregulation of these processes, together with the potential therapeutic role of curcumin in this context. We also summarized the molecular pathways involved in telomere maintenance, examined the influence of curcumin on these pathways, and proposed the potential therapeutic benefits of curcumin as a modulator of these molecular pathways.

The overall anticancer properties of curcumin are well documented. This review exclusively consolidates the evidence of its precise telomerase-inhibitory mechanisms, including hTERT transcriptional suppression, G-quadruplex stabilization, and chaperone disruption, in comparison with synthetic telomerase inhibitors.

We performed a comprehensive literature search across Google Scholar, PubMed, Web of Science, and Scopus databases. The search utilized keywords including: “telomeres,” “telomerase,” “curcumin,” “curcumin analogues,” “nanocurcumin,” and “cancer.” Of the more than 100 articles initially retrieved, 120 were included in this review based on their relevance and scientific quality. The final literature search was conducted on 30 September 2025.

## 2. Telomeres

**Figure 1:**
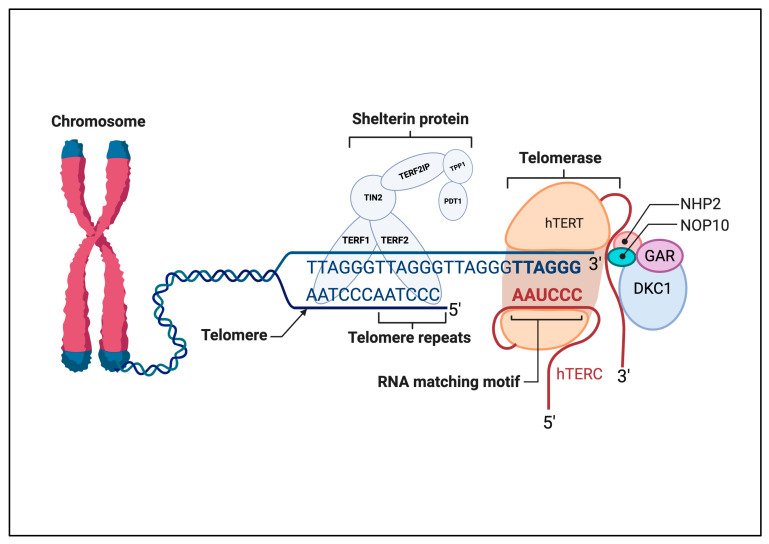
Schematic illustration of the interaction between telomerase and the telomere region at the very end of chromosomes. Telomerase is a ribonucleoprotein enzyme complex, with one molecule of telomerase reverse transcriptase (hTERT) and one molecule of telomerase RNA component (hTERC) serving as a template for the synthesis of the telomeric DNA repeat GGTTAG. Shelterin proteins TRF1, TRF2, TIN2, POT1, TPP1, and RAP1 bind to the telomeric DNA to cap the telomeres and maintain telomere length. The RNA templating motif of hTERC is aligned with the telomere repeats to allow base pairing for the extension of the telomere by hTERT. Other associated proteins, such as NHP2, NOP10, GAR, and DKC1, contribute to the stability and activity of the telomerase complex. This figure outlines the key components and interactions crucial for telomere maintenance and stability. Abbreviations: DKC1, dyskerin; hTERC, human telomerase RNA component; hTERT, human telomerase reverse transcriptase; POT1, protection of telomeres 1; RAP1, repressor activator protein 1; TCAB1, telomerase Cajal body protein 1; TIN2, TRF1-interacting nuclear factor 2; TPP1, TINT1/PTOP/PIP1; TRF1/2, telomere repeat-binding factor 1/2. Created in BioRender. ALBADRANI, h. (2025) https://BioRender.com/k16a761 (accessed on 17 September 2025).

Telomeres are sequences of repetitive non-coding nucleotides that are rich in guanine nucleotides (GGTTAG in humans). This sequence is attached to a protein complex known as shelterin, which involves six protein subunits: repressor activator protein (RAP1), telomere repeat binding factors 1 and 2 (TRF1 and TRF2), TRF1-interacting nuclear factor 2 (TIN2), protection of telomeres-1 (POT1), and TINT1/PTOP/PIP1 (TPP1) (Figure 1) [7]. This specialized structure is found at the terminus of linear chromosomes, where it forms a protective cap that regulates telomere metabolism, prevents chromatids from being recognized as double-stranded breaks, and is inappropriately repaired by fusion or degradation [8]. Telomeres participate in various cellular activities, including protecting chromosome ends, facilitating DNA replication, and regulating the cell cycle, thus helping to determine the lifespan of somatic cells by acting as a biological clock [9].

Telomeres play a crucial role in preserving genetic information during DNA replication by acting as buffer zones that ensure the completion of DNA synthesis without compromising chromosomal integrity. Moreover, telomeres maintain the correct separation of chromosomes during cell division and protect chromosomes from degradation, thus preventing the formation of aneuploid cells [10]. A typical cycle involves shortening of the telomere, with approximately 50–200 bases being lost from the sequence at the end of the chromosomes after each division until the telomere reaches a certain length, at which point the replication ability of the chromosome is lost, triggering cellular suicide [11]. The delicate balance between telomere shortening and telomerase activity is crucial for cellular health, as it ensures proper cell function and longevity. Disruption of this balance can lead to age-related diseases or carcinogenesis, in which telomerase is often reactivated to sustain uncontrolled cell proliferation. When the telomere becomes short, the response to DNA damage may be activated, which leads the cells to enter senescence or undergo apoptosis, preventing tumor formation [12]. In most cases, this barrier is overcome by upregulating telomerase activity or activating alternative telomere-lengthening mechanisms, allowing limitless proliferation and malignant transformation [13]. Incomplete or absent telomere function in cancer cells leads to genomic instability, which has significant consequences for tumor heterogeneity, metastasis, and therapeutic resistance, underscoring the importance of telomere regulation in cancer biology [14].

## 3. Telomerase and Cancer Progression

Telomerase is a specialized reverse transcriptase that increases telomere length by the addition of repetitive sequences (GGTTAG) to the ends of the telomeric region, counteracting the gradual shortening of telomeres that occurs with each round of cell division [15]. This enzyme complex is made up of two essential subunits and several complementary proteins: the integral human telomerase RNA component (hTERC), which acts as an RNA template, and the catalytic subunit of the hTERT (Figure 1). The protein complex comprises dyskerin (DKC1), telomerase Cajal body protein 1 (TCAP 1), NHP2, NOP10, and GAR, and has an important role in the modification and stabilization of specific uridine sequences in newly synthesized ribosomal RNA [15]. The main role of hTERT is to catalyze the addition of repetitive sequences to the RNA template. During telomeric elongation, hTERT binds to hTERC, which acts as a template, and the other complementary protein complex helps to localize and stabilize telomerase on the telomere (Figure 1). TCAP 1 is involved in recruiting hTERT to the telomere, whereas DKC1, NHP2, NOP10, and GAR1 stabilize binding during this process [16,17,18]. The G-quadruplex is a non-canonical, four-stranded secondary structure formed by nucleic acids, particularly in guanine-rich regions. Often located in telomeres, these structures play a protective role by capping chromosome ends and regulating telomerase activity [19].

In somatic cells, the activity of telomerase is tightly regulated and is inactive in most normal cells. However, it remains active in some tissues that require preservation of the telomere length and ensures ongoing regeneration and reproductive capacity [20,21,22]. However, telomerase dysregulation contributes to cancer development and enables cancer cells to maintain telomere length, allowing for indefinite proliferation and bypassing cellular senescence. This dysregulation can involve mutations in the TERT promoter region, epigenetic modifications, or alterations in signaling pathway activity (e.g., in the c-Myc and Wnt/β-catenin pathways), upregulating telomerase activity in cancer cells. Telomerase activity is upregulated in 90% of cancers and immortalized cells [23,24]. Elevated telomerase activity is linked to more aggressive tumor phenotypes, greater metastatic potential, and poor resistance to conventional cancer therapies. In esophageal cancer, the administration of low-dose chemotherapy or radiotherapy can accelerate the malignant phenotypic transformation in normal fibroblasts, leading to the activation of cancer-associated fibroblasts and increased tumor malignancy [25]. Mutations in telomerase and ATRX are independent predictors of poor prognosis in pheochromocytomas and paragangliomas, resulting in worse overall and metastasis-free survival [26]. Telomerase activity is significantly higher in breast cancer tissues than in normal tissues. Its activity is positively correlated with histological grade, tumor size, axillary nodal status, and the expression of both Ki-67 and HER-2/neu protein, and negatively correlated with estrogen receptor expression [27]. Studies have indicated that telomerase activation is linked to aggressive tumor behavior, heightened potential for metastasis, and limited efficacy of conventional cancer treatments.

## 4. Molecular Mechanisms of Telomer Elongation Contribute to Tumor Progression

### 4.1. Telomerase Activation Mechanism

Cancer cells stimulate various mechanisms to upregulate telomerase activity, thereby preserving telomere length and sustaining proliferation. One key mechanism involves mutations in the non-coding regions of the hTERT promoter, which create ETS factor-binding sites that lead to transcriptional hyperactivation of telomerase [28]. Additionally, viral oncoproteins can interact with the regulatory elements of host cells, further enhancing TERT gene activation [29]. hTERT expression is elevated in cancer cells, resulting in higher telomerase activity, whereas in normal cells, hTERT expression remains suppressed [30]. Epigenetic modifications, such as histone modifications, DNA methylation, and non-coding RNAs, also regulate telomerase by influencing TERT gene expression [31]. Most tumor cells exhibit increased hTERT promoter hypermethylation, which is associated with tumorigenesis. In contrast, telomerase-negative normal tissues exhibit hypomethylation [32,33]. The histone methyltransferase SMYD3 adds another layer of regulation by binding to the TERT promoter and activating its transcription through the methylation of histone H3K4 in both fibroblasts and cancer cells [34]. The TERT promoter commonly exhibits histone modifications; for example, the active histone marks H3K27ac and H3K4me3 and epigenetic silencing marks H3K9me3 and H3K27me3. H3K4me3, which is linked to active transcription, is significantly enriched in induced pluripotent stem cells and cancer cells. In contrast, HP-1α and H3K27me3, which form heterochromatin, are highly enriched in somatic cells. Additionally, numerous non-coding RNAs bind to TERT, including various microRNAs (miRNAs) that target the 3′-untranslated regions (3′-UTRs) and open reading frames of TERT, thereby modulating its expression in various cancer cell types [35]. miRNAs can modulate transcription by inhibiting transcription factors associated with TERT.

### 4.2. Curcumin Suppresses Telomerase Activity by Modulating Specific microRNAs

**Figure 2:**
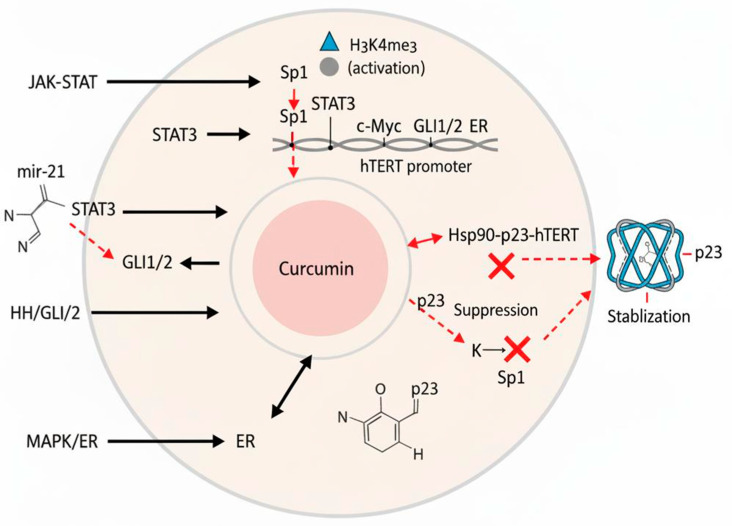
Diagram illustrating the regulation of hTERT (human telomerase reverse transcriptase) and curcumin’s multifaceted inhibitory mechanisms. Transcription factors (Sp1, STAT3, c-Myc, GLI1/2, and ER), epigenetic modifications (H3K4me3 activation and H3K27me3 repression), and miRNAs (e.g., miR-21) modulate hTERT promoter activity. Curcumin inhibits telomerase by disrupting the Hsp90–p23–hTERT complex, suppressing Sp1/STAT3 activity and expression, and stabilizing the telomeric G-quadruplex DNA structure. Abbreviations: ER, estrogen receptor; G4, G-quadruplex DNA; hTERT, human telomerase reverse transcriptase; Hsp90, heat shock protein 90; miRNA, microRNA; Sp1, specificity protein 1; STAT3, signal transducer and activator of transcription 3.

This miRNA-mediated mechanism represents a promising pathway for telomerase inhibition. Additionally, the antitumor activity of curcumin in osteosarcoma involves miR-138, which has been shown to inhibit cell proliferation and invasion [36], highlighting the broader role of miRNA regulation in its mechanism of action. A review of the literature has suggested that miR-135-5p may mediate curcumin’s anti-telomerase effects in ovarian cancer [37]. Furthermore, other microRNAs, such as miR-21, also regulate TERT by targeting different pathways, including STAT3 signaling in glioblastoma and the PTEN/ERK1/2 pathway in colorectal cancer (Figure 2) [38,39].

Some studies have explored the role of TERT in both breast and ovarian cancers, highlighting its promise as a therapeutic target. Wang et al. assessed hTERT function in ovarian cancer using the SKOV3 cell line. Their findings showed that reducing hTERT led to a higher rate of apoptosis and a decline in cell proliferation [40]. Similarly, Su et al. [41] employed a mouse transplantation tumor model and found that lowering hTERT levels significantly suppressed ovarian cancer growth by enhancing apoptosis and upregulating senescence-associated genes, such as p21 and p53, which are essential for controlling cell cycle and apoptosis [41]. Additionally, notable progress has been made in developing breast cancer treatments that focus on targeting hTERT G-quadruplex (G4) structures. Researchers have focused on designing small-molecule ligands that specifically bind to the G4 structures, aiming to downregulate hTERT expression [42].

In cancer cells, telomerase activity is stimulated by certain signaling pathways. The JAK-STAT pathway, which plays a significant role in activating telomerase in cancer cells [43]. In hematological malignancies, the JAK-STAT pathway is frequently upregulated, contributing to telomerase activation [43]. The JAK-STAT pathway mediates signaling through growth factors and cytokine receptors, and its activation leads to the phosphorylation of JAK and STAT proteins [44]. Phosphorylated STAT proteins bind directly to the hTERT promoter, resulting in telomerase activation [43]. Additionally, the JAK-STAT pathway is linked to the mTORC1/PI3K/HSP90/AKT pathway, which regulates telomerase activity [45].

Furthermore, the Hedgehog (HH)/GLI axis influences telomerase activity by directly targeting the hTERT gene in cancers [46,47]. Activation of the HH/GLI signaling pathway increases the production of the GLI1 and GLI2 transcription factors, which attach to the hTERT promoter and stimulate its transcription [48]. Inhibiting the function of GLI1/GLI2 using a pharmacological inhibitor or a truncated GLI3 repressor mutant led to decreased protein levels of hTERT and reduced telomerase activity in various cancer cell lines [47,49]. Conversely, introducing a constantly active GLI2 mutant resulted in elevated mRNA and protein levels, as well as increased hTERT promoter activity (Figure 2) [50].

Moreover, androgen receptor (AR)-mediated signaling upregulates telomerase expression in cancer cells. In androgen-dependent prostate cancer cells, ARs positively regulate hTERT expression [51]. In contrast, in castration-resistant prostate cancer cells, AR-mediated signaling negatively regulates hTERT expression [52]. This negative regulation involves the transcription factor EGR1, which positively regulates hTERT expression [53].

In endometrial cancer cells, the MAPK pathway also contributes to the stimulation of estrogen-mediated induction of telomerase activity [54]. When cells are exposed to estrogen, the MAPK pathway, specifically the p44/42 MAPK, ERK1/2, and P38 members, is activated, which in turn regulates telomerase activity [54,55,56,57,58]. Estrogen-driven MAPK activation results in the phosphorylation of hTERT, the enzyme’s catalytic subunit, and promotes the association of 14-3-3 protein and NF-κB with hTERT. Additionally, the MAPK pathway is involved in the transcriptional regulation of hTERT through activation of the hTERT promoter, which contains estrogen-responsive elements. The MAPK family members JNK, ERK1/2, and P38 play distinct roles in regulating hTERT levels and telomerase activity. Ultimately, radiation stimulates telomerase activity in cancer cells through the Ras/phosphatidylinositol 3-kinase/Akt pathway [59].

### 4.3. Mechanism of Alternative Lengthening of Telomeres

Telomeres can also elongate through telomerase-independent mechanisms, such as alternative lengthening of telomeres (ALT). In this process, telomere length is maintained by homologous recombination between telomeric DNA sequences. This process takes place when the telomere is absent and is often triggered by defects in the telomere maintenance machinery, including mutations in the chromatin remodeling complex (DAXX/ATRX), which are commonly found in certain cancer types [31]. This alternative pathway is often observed in cancers that manifest telomerase activity or in tumors with mutations that increase telomerase function. Specific genomic alterations and histological features are associated with ALT activation in various cancers. ALT activation is characterized by heterogeneous telomere lengths, extrachromosomal telomeric DNA circles, and specialized nuclear structures known as ALT-associated PML bodies. However, it is unclear how ALT is activated in cancer cells at a molecular level. However, several key factors that trigger the ATL mechanism in cancer cells have been identified [60]. One of the primary triggers is loss of function or mutations in the ATRX/DAXX chromatin remodeling complex, which is common in tumors with ALT. Homologous recombination proteins, such as RAD52, are involved in one of the break-induced replications (BIR) that are the basis of ALT activation. Other key BIR events involve the fork remodeler SMARCAL1 and nuclease MUS81, which maintain replication fork stability and encourage DNA repair [61]. Another key contributor to this alternative lengthening method is the activation of the DNA damage response, which promotes telomere elongation via homologous recombination. In addition, somatic mutations or dysregulation of telomere maintenance mechanisms can trigger bypass of the ALT pathway, allowing cancer cells to preserve telomere length through normal telomerase activity [62]. This constitutes an unequivocal avoidance of cellular senescence, which allows continuous proliferation.

The ALT pathway is also associated with two distinct BIR processes: one dependent on RAD52 and the other dependent on the fork remodelers SMARCAL1 and MUS81 nuclease [61]. Furthermore, the ALT pathway involves telomerase activation or activation of TMMs through reactivation of telomerase activity [62]. These molecular mechanisms provide insights into the processes involving ALT and potential therapeutic targets for the ALT pathway in cancer cells [63].

## 5. Telomerase and Curcumin

Curcumin, widely recognized as turmeric, is a natural yellow polyphenolic compound isolated from *Curcuma longa*. Curcumin has been used in alternative medicine for many years as a compound with medicinal properties [64]. Curcumin is chemically composed of diferuloyl methane, which consists of two ferulic acid residues joined by a methylene bridge to yield a bright yellow pigment. This bioactive compound exists in different forms, including free curcumin, curcumin complexes, and derivatives such as tetrahydrocurcumin, all of which have different bioavailabilities and therapeutic potentials [65].

Turmeric has been used for its putative healing properties in skin, pulmonary, and gastrointestinal ailments, and for the treatment of wounds and sprains. Curiously, many of these traditional uses have been verified in recent studies that have investigated the cytoprotective mechanisms of curcumin in several pro-inflammatory diseases. The therapeutic benefits of curcumin are due to its anti-inflammatory and antioxidant properties [66].

One of the most promising areas of research is the potential use of curcumin as an anticancer agent. Curcumin can induce programmed cell death and apoptosis in cancer cells, inhibit the proliferation of cancer cells, and inhibit the diffuse growth of tumor cells. Several studies have demonstrated that curcumin effectively inhibits the growth of colorectal cancer cells and has also shown promising results in breast and pancreatic cancers [67,68,69]. These results underscore the notable promise of curcumin in cancer therapy, making it a focal point of ongoing medical research.

The mechanism by which curcumin inhibits telomerase is fundamentally different from that of synthetic agents. Unlike imetelstat, which directly blocks the hTERC RNA template, curcumin epigenetically silenced hTERT. This hypothesis is supported by two lines of evidence: first, the hTERT subunit is known to be regulated by epigenetic mechanisms, including DNA methylation [70], and second, curcumin has been demonstrated to function as a DNA-demethylating agent, as shown by its ability to reactivate silenced tumor suppressor genes in lung cancer models [71]. This epigenetic activity acts in concert with curcumin’s ability to disrupt key transcription factor networks, such as Sp1, STAT3, and c-Myc, to achieve multitargeted inhibition of telomerase.

## 6. Mechanisms of Curcumin-Mediated Telomerase Regulation

Curcumin suppresses the activity of telomerase, leading to apoptosis and shortening of telomeres in various cancer cell types, including brain tumor [9], human leukemia [72,73], and MCF-7 breast cancer [6].

Telomerase activity is reactivated in immortal cells and in most human malignancies. Curcumin suppresses telomerase activity and reduces hTERT mRNA levels, which leads to telomere shortening of telomere [9]. Telomerase activity is extremely high in cancer cells. Curcumin has been shown to reduce telomerase levels in human leukemia cells by 55% and 78% after 24 h of treatment with 10 and 50 mM curcumin, respectively [73], suggesting that curcumin inhibits the translocation of hTERT from the nucleus to the cytosol. Nuclear localization of hTERT involves its interaction with the Hsp90-p23 complex. Curcumin disrupts this interaction by dissociating p23 from hTERT, leading to its cytoplasmic accumulation, subsequent proteasomal degradation, and ubiquitination, while leaving the binding of Hsp90 to hTERT unaffected [74].

Khaw and colleagues investigated the effects of curcumin on telomere shortening and telomerase activity in human glioblastoma cell lines. Curcumin was administered at concentrations ranging from 0 to 100 µM, demonstrating a dose-dependent effect on cell viability. Curcumin downregulates hTERT mRNA expression and inhibits telomerase activity, resulting in telomere shortening in these cancer cells. This inhibition resulted in growth arrest at the G2/M phase and induced apoptosis via increased caspase-3/7 activity and overexpression of pro-apoptotic proteins, such as Bax, while decreasing the expression of anti-apoptotic proteins, such as Bcl-2 [9]. Nanoencapsulation of curcumin increases its delivery to cancerous cells, leading to a significant decrease in hTERT gene expression [75,76]. This approach is further supported by recent in vivo and clinical evidence. For instance, reviews on nutraceutical telomerase inhibitors and nanocurcumin formulations have shown that PLGA–curcumin nanoparticles can downregulate hTERT expression and shorten telomeres in colorectal tumors [77,78,79]. However, direct validation in well-controlled primary studies is essential.

The downregulation of hTERT mRNA expression occurs via different mechanisms. First, it generates ROS, which eventually inhibits Sp1 binding activity, thereby downregulating hTERT expression [80]. Furthermore, curcumin reduces the expression of Sp1, a major transcription factor that regulates hTERT, via a proteasomal mechanism, ultimately leading to the inhibition of hTERT mRNA levels [80]. Moreover, curcumin’s ability to downregulate DNMT3b mRNA levels contributes to the suppression of hTERT levels, as demonstrated in lung cancer cells, revealing a novel molecular pathway by which curcumin may act as a chemopreventive agent in lung cancer involving the reactivation of tumor suppressor genes such as RARβ, which ultimately impacts hTERT expression [71]. Collectively, these findings suggest that curcumin exerts its anticancer effects, in part, by downregulating hTERT mRNA expression through multiple pathways.

Curcumin interacts with telomeric G-quadruplexes and inhibits telomerase activity [19]. This interaction involves groove binding to G-quadruplex DNA, and curcumin has been shown to binds strongly to these structures. Curcumin also affects other telomere-associated proteins. Curcumin reduces the expression of telomerase-associated protein 1, which is essential for the stability and activity of the telomerase complex. The downregulation of TEP1 expression is due to the reduced expression of protein arginine methyltransferase 5, a key enzyme that participates in histone methylation and modulates the expression of TEP1 and other proteins involved in telomerase regulation [81].

Curcumin indirectly inhibits telomerase activity by modifying several cellular signaling pathways, including its selective targets on proteins associated with the regulation of cell cycle control. For example, curcumin induces cell cycle arrest in the G1 phase by targeting cyclin-dependent kinase 2 (CDK2) activity, thereby suppressing cancer cell proliferation [82]. Additionally, curcumin analogs, such as chemoprevention curcumin analog-1.1 (CCA-1.1) and pentagamavunone-1 (PGV-1), induce mitotic arrest and cellular senescence in hepatocellular carcinoma cells, further emphasizing the significance of curcumin derivatives in cell cycle regulation [83,84]. Chemically modified curcumin (mCur) induces multiphase cell cycle arrest, including G2/M phase arrest, by downregulating cyclin-dependent kinases and polo-like kinase 1 (PLK1), CCNE1, E2F1, and CDK2, while enhancing PTEN gene expression. Xu et al. used colorectal cancer cell lines to evaluate the impact of curcumin nanoprodrugs on cell cycle progression. The suppression of CDKs and downregulation of PLK1 may contribute to cellular stress and growth arrest, which can indirectly affect telomere maintenance [85]. In addition, curcumin triggers senescence by inducing mitotic slippage, DNA damage, and p21waf1/cip1-dependent heterochromatin loss in cancer cells [86]. Curcumin enhances DLC1, a tumor suppressor gene that inhibits the DYRK1A-EGFR axis, triggers DNA damage, and ultimately leads to cancer cell senescence [87]. These findings collectively highlight the multifaceted molecular pathways through which curcumin exerts its senescence-inducing effects on cancer cells while affecting the cell cycle and telomerase activity.

## 7. Mechanistic Pathways

Curcumin exerts multiple anticancer effects by modulating key oncogenic signaling pathways, including JAK/STAT, MAPK/ERK, the androgen receptor axis, and PI3K/AKT [37,88]. It also regulates microRNAs involved in telomerase control [37,89].

Curcumin directly inhibits the JAK2/STAT signaling cascade, inducing apoptosis in JAK2-mutated cells through suppression of this pathway [90]. This finding is further supported by evidence from gastric cancer models, where curcumin suppresses JAK2/STAT signaling to overcome chemoresistance [91].

It suppresses the PI3K/AKT pathway by restoring PTEN expression, leading to a 60% decrease in p-AKT (Ser473) levels and downstream mTOR signaling [36,37,92]. Curcumin decreases p-ERK1/2 levels, resulting in the inhibition of ELK1-driven hTERT transcription and reduced GLI1 and PTCH1 transcripts within the hedgehog/GLI pathway [71,75,92]. It also inhibits AKT-dependent AR phosphorylation, reducing AR nuclear localization and PSA expression in prostate cancer cells by approximately 60% [51,93,94].

Curcumin inhibits telomerase by reducing oncogenic microRNA-21 levels by 50%, thereby relieving the suppression of PTEN and SOCS3, which in turn decreases AKT and STAT3 signaling [38,95]. Conversely, it increases miR-138, a tumor-suppressive microRNA that directly targets the 3′ UTR of hTERT mRNA, leading to an approximately 50% reduction in telomerase activity, an effect confirmed in osteosarcoma, glioblastoma, and ovarian cancer models, where blocking miR-138 attenuates curcumin’s anti-telomerase action [89,95]. Additionally, miR-21 influences hTERT regulation via the STAT3 and PTEN/ERK1/2 pathways, linking curcumin-mediated modulation of miRNAs to reduced telomerase expression and telomere shortening [37,38,95]. Together, these results demonstrate that curcumin impedes cancer cell immortality by inhibiting oncogenic signaling and telomerase via a microRNA-dependent mechanism [37,71,92].

## 8. Comparative Perspective

The broad, multitargeted modulatory effects of curcumin on telomerase differ from those of synthetic inhibitors, such as imetelstat, the most extensively studied direct telomerase antagonist [96]. Imetelstat prevents telomeric DNA extension by binding to the telomerase RNA template. However, due to dose-related hematologic toxicity and the emergence of resistance mechanisms, its clinical use remains limited despite its efficacy against hematologic malignancies [97,98].

In contrast, curcumin employs a pleiotropic strategy: inhibiting telomerase through both transcriptional and post-translational mechanisms, stabilizing telomeric G-quadruplexes, suppressing pro-inflammatory signaling, and exerting antioxidant activity [77,99,100]. This polypharmacological profile may lower the likelihood of resistance and enhance the potential for synergistic therapy, although it simultaneously poses challenges for standardization and clinical application [93,99].

## 9. Challenges and Future Directions

### 9.1. Overcoming Pharmacokinetic Limitations

The clinical application of curcumin is hindered primarily by its poor pharmacokinetic properties, including low water solubility, rapid metabolism, and rapid systemic elimination [93,99,101]. When administered orally, curcumin undergoes extensive first-pass metabolism in the liver, producing metabolites that are swiftly cleared from the bloodstream [93,99]. These pharmacokinetic barriers limit the achievement of therapeutically effective drug concentrations in the target tissues. Consequently, as highlighted in recent reviews of clinical trials, substantial research has focused on overcoming these limitations by using advanced formulation strategies. Karaboga Arslan et al. (2022) reported the use of innovative delivery systems, such as phytosomal complexes (e.g., Meriva^®^) and nanoparticle-based formulations (e.g., Theracurmin^®^), which enhance curcumin’s bioavailability and therapeutic efficacy [102].

### 9.2. Promising Strategies: Analogs and Delivery System

**Figure 3:**
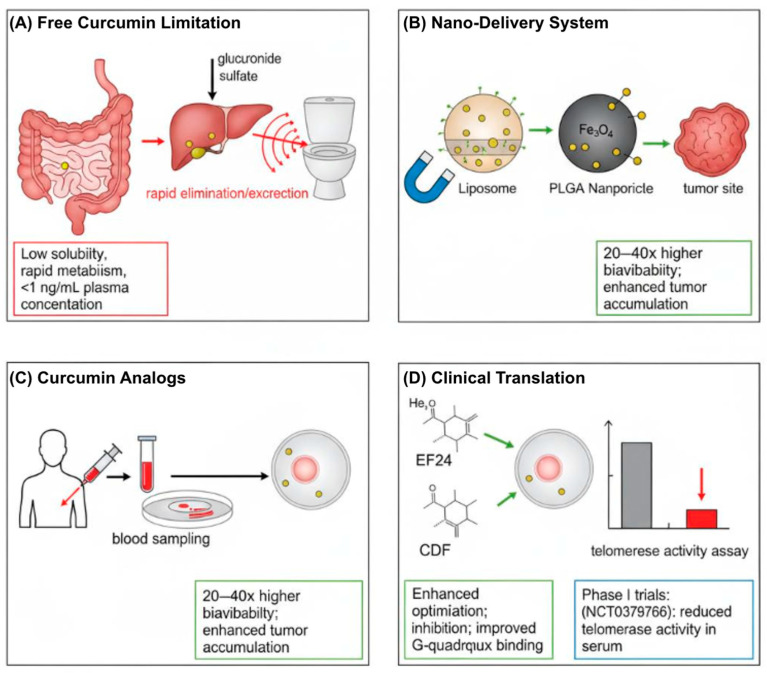
Strategies to overcome the bioavailability limitations of curcumin for telomerase-targeted cancer therapy. (**A**) Free curcumin exhibits poor solubility, rapid hepatic metabolism, and rapid systemic clearance, resulting in subtherapeutic plasma concentrations. (**B**) Nanoformulations (e.g., liposomes, PLGA nanoparticles, and metal–curcumin complexes) enhance solubility, prolong circulation time, and enable tumor-targeted delivery. (**C**) Structurally optimized analogs (e.g., EF24, CDF) demonstrate superior hTERT inhibition and G-quadruplex stabilization. (**D**) Potential clinical outcome: nano-curcumin-mediated suppression of telomerase activity in patient serum. Arrows in the diagram generally indicate processes, pathways, or transitions between states. Abbreviations: CDF, difluorinated curcumin; G4, G-quadruplex DNA; hTERT, human telomerase reverse transcriptase; PLGA, poly(lactic-co-glycolic acid).

To address these limitations, research has focused on synthesizing curcumin analogs and developing advanced delivery systems (Figure 3). Analogs such as CCA-1.1, PGV-1, and EF24 have demonstrated improved potency, stability, and bioavailability [103,104]. Notably, the difluorinated curcumin analog (CDF) exhibits 10–30-fold greater hTERT inhibition than native curcumin, primarily through the suppression of STAT3 phosphorylation [105]. Similarly, the synthetic analog EF24 shows potent telomerase inhibition in glioblastoma xenografts by suppressing Sp1-mediated hTERT transcription [106].

Beyond organic derivatives, metal–curcumin complexes represent another promising avenue. For instance, in colorectal cancer models, an aluminum–curcumin complex demonstrated superior potency compared to native curcumin [107]. Collectively, the development of such enhanced analogs and metal–curcumin complexes constitutes an actively pursued strategy to overcome the bioavailability and efficacy limitations of native curcumin [103,108].

To circumvent these challenges, a great deal of research has been dedicated to the synthesis of curcumin analogs and their delivery systems. Analogs and Derivatives: Compounds such as CCA-1.1, PGV-1 and EF24 show increased stability and efficiency [83,104]. For example, the curcumin analog CDF (difluorinated curcumin) displays 10–30× more potent hTERT suppression than the precursor compound curcumin through the suppression of STAT3 phosphorylation and Sp1 nuclear translocation [71] grafts by suppressing Sp1-mediated hTERT transcription [105,106].

Nanotechnological approaches—including polymeric nanoparticles [101,108,109], liposomal or micellar systems [110,111,112], and other advanced nanocarriers—have markedly improved curcumin’s bioavailability, metabolic stability, and tumor-targeting efficiency [113,114]. Recent preclinical and systematic reviews have confirmed these benefits [78], and several early-phase clinical trials are currently evaluating these formulations in patients [115].

A Phase I clinical trial demonstrated the safety and feasibility of administering liposomal curcumin (LipoCurc) to patients with malignant pleural effusion [115]. Preclinical studies have indicated that dendrosomal and solid lipid nanocurcumin formulations enhance bioavailability and anticancer activity in vitro. For example, dendrosomal nanocurcumin decreased telomerase activity via the TGF-β1 pathway in hepatocellular carcinoma cells [92], whereas curcumin-loaded OA400 nanoparticles suppressed HPV oncogene expression in cervical cancer cells [116]. Furthermore, PLGA-based nanoparticles have been investigated for the treatment of malignant pleural effusion [115] and several other cancers [102]; however, direct evidence of telomerase inhibition in human trials remains limited.

Encouraging findings from advanced formulations have also emerged from clinical studies. For instance, a Phase II trial reported improved disease control rates and median overall survival using a phytosomal curcumin formulation combined with gemcitabine in pancreatic cancer [84]. Preclinical PLGA-based curcumin nanoparticles showed increased tumor accumulation associated with hTERT downregulation in experimental models [117]. In a pilot clinical study, oral curcumin improved oxidative status in prostate cancer patients [20]; however, robust clinical evidence demonstrating significant effects on leukocyte telomere length and telomerase activity is still lacking.

### 9.3. Comparative Analysis of Telomerase Inhibitors

Comparative evaluation indicated that the multitargeted mode of action of curcumin differs from that of synthetic agents such as BIBR1532, a direct telomerase catalytic inhibitor, or G-quadruplex stabilizers. Although these agents exhibit higher potency in specific contexts, curcumin offers a broader safety margin, pleiotropic efficacy across diverse tumor types, and a favorable potential for combination therapy [93,99,118]. Nevertheless, its naturally low bioavailability remains a major challenge compared to that of certain synthetic inhibitors [39,78,108] (Figure 3). Table 1 summarizes the key characteristics of representative telomerase inhibitors to contextualize these differences.

### 9.4. Future Research

Future investigations should prioritize well-designed in vivo studies that directly assess telomere length and telomerase activity as primary endpoints. Developing a standardized bioavailable curcumin formulation is essential for successful clinical translation (Figure 3) [39,78,108]. Clinical trials should incorporate telomere-related biomarkers to accurately evaluate the potential of curcumin as a telomerase-targeted therapy [77,92]. Additionally, exploring combination strategies with standard chemotherapeutics or other telomerase inhibitors may yield synergistic anticancer effects [93,96]. This synergism may extend to natural compounds such as piperine, which enhances curcumin’s bioavailability and efficacy [119]. Furthermore, curcumin’s potential role as a geroprotective agent against age-related diseases, including cancer, through telomere maintenance warrants further investigation [120].

## 10. Conclusions

This review highlights the pivotal role of telomeres and telomerase in tumor progression, emphasizing the complex regulatory mechanisms that enable tumor cells to evade senescence, either through telomerase reactivation or alternative lengthening of telomeres (ALT). The unlimited replication potential, genomic instability, and resistance to conventional therapy underscore telomere maintenance as a compelling therapeutic target.

Curcumin has emerged as a promising natural modulator of this axis. It downregulates hTERT expression, disrupts telomerase assembly (e.g., interference with the Hsp90–hTERT complex), stabilizes telomeric G-quadruplex structures, and modulates key oncogenic signaling pathways, including JAK/STAT, NF-κB, and Wnt/β-catenin. Collectively, these effects induce cell-cycle arrest, senescence, and apoptosis in multiple cancer types.

However, translating these robust preclinical outcomes remains constrained by pharmacokinetic limitations, including rapid hepatic and intestinal degradation, hydrophobicity, and low systemic bioavailability. To address these challenges, several measures are critical: (1) application of nanotechnology to enhance solubility, slow metabolic degradation, and increase serum concentration; (2) design of optimized derivatives with high selectivity and affinity for telomerase-associated molecular targets; and (3) rigorous, biomarker-driven clinical trials using standardized protocols to evaluate efficacy, safety, and patient stratification based on telomerase activity or ALT pathway activation.

Through the integration of these strategies, curcumin may evolve from a promising phytochemical into a clinically viable telomerase-modulating agent. Its mechanistic multi-targeted activity across the telomere–telomerase axis offers distinct advantages over synthetic inhibitors that typically act at a single molecular site. Ultimately, curcumin-based interventions present an opportunity to develop innovative therapeutic approaches to improve outcomes across diverse malignancies.

## Figures and Tables

**Table 1:** Comparative profile of telomerase inhibitors in oncology.

Inhibitor	Mechanism of Action	Bioavailability	Cancer Types Tested	Clinical Stage
Curcumin	hTERT transcription; G-quadruplex stabilization; Hsp90-p23 disruption	Very low (<1 ng/mL)	Prostate Cancer	Phase I/II (NCT03769766) *
Liposomal curcumin	Enhanced tumor delivery; sustained hTERT suppression	20–40× higher than free curcumin	Colorectal, pancreatic	Phase I
Imetelstat (GRN163L)	hTERC template antagonist (antisense oligonucleotide)	Moderate (IV only)	Myelofibrosis, AML	Phase III
BIBR1532	Non-nucleoside hTERT catalytic inhibitor	Low (preclinical)	Prostate, lung	Preclinical
EF24 (curcumin analog)	STAT3/Sp1 inhibition; ROS-mediated hTERT downregulation	Moderate (nanoparticle-formulated)	Triple-negative breast cancer	Preclinical

* The mechanism in this clinical trial is not directly confirmed [102].

## Abbreviations

ALT	alternative lengthening of telomeres
AR	androgen receptor
CCA-1.1	chemoprevention curcumin analog-1.1
CDF	difluorinated curcumin
c-Myc	cellular myelocytomatosis oncogene
DKC1	dyskerin
EF24	curcumin analog EF24
ER	estrogen receptor
G4	G-quadruplex DNA
GLI1/GLI2	GLI family zinc-finger 1/2
H3K4me3	histone 3 lysine 4 trimethylation
H3K27me3	histone 3 lysine 27 trimethylation
Hsp90	heat shock protein 90
hTERC	human telomerase RNA component
hTERT	human telomerase reverse transcriptase
JAK/STAT	Janus kinase/signal transducer and activator of transcription
MAPK	mitogen-activated protein kinase
mCur	chemically modified curcumin
miRNAs	microRNAs
PGV-1	pentagamavunone-1
PI3K/AKT	phosphoinositide 3-kinase/protein kinase B pathway
PLGA	poly(lactic-co-glycolic acid)
PLK1	polo-like kinase 1
POT1	protection of telomeres 1
RAP1	repressor activator protein 1
SMYD3	set and mynd domain-containing protein 3
Sp1	specificity protein 1
STAT3	signal transducer and activator of transcription 3
TCAB1	telomerase Cajal body protein 1
TEP1	telomerase-associated protein 1
TIN2	TRF1-interacting nuclear factor 2
TPP1	TINT1/PTOP/PIP1 (protection of telomeres 1-interacting protein)
TRF1/2	telomere repeat-binding factors 1 and 2
